# Polymicrobial Bloodstream Infection in Neonates: Microbiology, Clinical Characteristics, and Risk Factors

**DOI:** 10.1371/journal.pone.0083082

**Published:** 2014-01-14

**Authors:** Ming-Horng Tsai, Shih-Ming Chu, Jen-Fu Hsu, Reyin Lien, Hsuan-Rong Huang, Ming-Chou Chiang, Ren-Huei Fu, Chiang-Wen Lee, Yhu-Chering Huang

**Affiliations:** 1 Division of Neonatology and Pediatric Hematology/Oncology, Department of Pediatrics, Chang Gung Memorial Hospital, Yunlin, Taiwan; 2 Division of Pediatric Infectious Disease, Department of Pediatrics, Chang Gung Memorial Hospital, Taoyuan, Taiwan; 3 Division of Pediatric Neonatology, Department of Pediatrics, Chang Gung Memorial Hospital, Taoyuan, Taiwan; 4 College of Medicine, Chang Gung University, Taoyuan, Taiwan; 5 Chang Gung University of Science and Technology, Chiayi, Taiwan; University of Cincinnati, United States of America

## Abstract

**Background:**

Polymicrobial bloodstream infections (PBSIs) have been associated with complex underlying medical conditions and a high incidence of specific microorganisms in several settings, but the relevant data are scarce in neonates.

**Methods:**

Positive blood cultures from January 2004 to December 2011 in the neonatal intensive care unit (NICU) of Chang Gung Memorial Hospital (CGMH) were reviewed. Each neonate with PBSI (case episode) was matched to two episodes of monomicrobial BSI (control episode) by birth weight, gestational age and gender. Records were reviewed to compare their underlying medical conditions, organisms isolated, adequacy of therapy, clinical characteristics and outcomes.

**Results:**

Forty-five episodes of PBSI (4.4% of all neonatal BSIs) were identified in 43 neonates. Gram-negative organisms constituted 59.8% of all PBSI pathogens, and 33 (73.3%) of PBSIs were caused by at least one Gram-negative organism. PBSIs were significantly more likely to be the recurrent episode and have endotracheal tube in place. No significant difference was found between PBSIs and controls in terms of demographics and most chronic conditions. PBSIs were significantly associated with a higher severity of illness, a longer duration of septic symptoms, and a higher rate of modification of antimicrobial regimens than monomicrobial BSIs. However, the sepsis-attributable mortality rates were comparable between these two groups.

**Conclusions:**

In the NICU, PBSIs were more often caused by Gram-negative bacilli, and often occurred in neonates without any chronic conditions. The clinical significance of PBSIs included a more severe illness, longer duration of septic symptoms and a higher rate of modification of antimicrobial regimens.

## Introduction

Polymicrobial bloodstream infection (PBSI) has been reported to be related to higher rates of various underlying chronic conditions and/or presence of medical devices in several different settings [1]–[5]. The prevalence of PBSI is reported to be around 5% to 27% of all bloodstream infections in adult or pediatric intensive care unit [1], [2], [4]–[8], and is found to be associated with a higher attributable mortality rate than monomicrobial BSIs [3], [9]–[11]. Appropriate treatment of PBSI are often more difficult since the identification of more than one bacterium usually takes more time [1], [11]. Several studies were conducted to address the issue, including which patients were at risk for PBSIs and special consideration should be given when initiating empirical antibiotics, but the results were inconsistent [1], [2], [12], [13].

Polymicrobial BSI is rarely described in the neonatal population, and only two matched case-control studies were reported in the literature to date [2], [13]. While Bizzarro *et al.* [2] concluded PBSI often occurred in neonates with severe underlying conditions and prolonged indwelling central venous catheter (CVC), these predisposing factors were not observed in Gupta *et al.*'s study [13]. Clinical characteristics and antimicrobial treatment for these neonatal PBSIs were not addressed in both studies. Besides, the impact of empiric antimicrobial regimens on clinical outcome of PBSI was not elucidated yet. In this study, we aimed to identify the risk factors for neonatal PBSI, and delineate the microbiology, clinical characteristics, antimicrobial therapy and clinical outcomes of neonates with PBSI.

## Materials and Methods

### Setting and Study Design

This study was conducted in the neonatal intensive care unit (NICU) of Chang Gung Memorial Hospital (CGMH), a tertiary level, university-affiliated teaching hospital in northern Taiwan. The NICU contains three units and has a total capacity of 49 beds equipped with mechanical ventilator and 28 beds with special care nurseries. There are more than 700 neonates admissions per year in our NICUs, and approximately one-third of them are outborn and transferred from other hospitals. A microbiological database search of all blood cultures obtained from patients of our NICUs between January 2004 and December 2011 was performed. This study was approved by the institutional review board of CGMH, with a waiver of informed consent.

Clinical data were analyzed by episodes, which were defined as distinct periods of clinical illness in association with positive blood cultures. All PBSI episodes (defined as case episodes) were those having 2 or more organisms isolated from one or more blood cultures within a 48-hour period. Episodes were considered distinct if separated by at least 7 days of clinical improvement and absence of positive blood cultures. During the same period, each case episode was matched to two control episodes of monomicrobial BSI by gender, birth weight and gestational age. Since the causative pathogens of neonatal BSI have predictable clinical features and outcomes [14]–[16], the pathogens of the monomicrobial BSI control episodes were arranged based on the pathogen distribution of overall cohort of neonatal BSIs. There were restrictions on the number of BSIs per patient that could be included as case or control episodes, which indicated a neonate with multiple episodes of BSI could be included in either the case or control group once.

### Microbiological methods

At our NICUs, a single blood culture is usually obtained peripherally (never through central venous catheter [CVC]) when an infant develops clinical symptoms and signs of sepsis. The blood culture system is Bactec 9240, and the volume of blood taken for each culture is at least 0.5 mL but usually 1.0 mL. Additional blood culture drawing or culture from other sterile sites depended on the discretion of each attending physician. The hospital's microbiology laboratory determined antimicrobial susceptibility of isolates according to definition of the National Committee for Clinical Laboratory Standards [17].

### Data collection

Charts and electronic records were reviewed to obtain demographic data, underlying conditions and all details of each case and control episode including the clinical features, laboratory data, treatment strategies and outcomes. The following comorbid conditions were also recorded: use of CVC, total parenteral nutrition (TPN) and/or intrafat, mechanical ventilators (conventional or high frequency osciallatory ventilator [HFOV]), and receipt of corticosteroid, any antibiotics, and surgical interventions within 30 days prior to bacteremia. Severity of illness was calculated by two investigators (Dr. S.-M.C. and Dr. J.-F.H.) at the first day of BSI based on the Neonatal Therapeutic Intervention Scoring System (NTISS) [18].

### Definitions

An episode of laboratory-confirmed BSI was defined as a recognized pathogen isolated from ≥1 blood culture in a patient with signs or symptoms of systemic illness (i.e., fever >38.0°C, apnea or respiratory distress, feeding intolerance, hypo- or hyperglycemia, hypotension, etc.). For the case episodes, the yielding of coagulase-negative staphylococci (CoNS), α-hemolytic streptococci, *Propionibacterium acnes*, *Corynebacterium* spp. and *Bacillus* spp. were considered contaminants unless the organism was identified from at least an additional culture from blood, catheter tip, or other sterile site. All possible contaminants and true pathogens enrolled as the case episodes were judged by an infectious disease consultant. For the control episodes and overall cohort, the definitions of BSI caused by CoNS and *Staphylococcus aureus* were according to the criteria from the Centers for Disease Control (CDC) and Prevention [19].

All comorbidities of prematurity, including respiratory distress syndrome (RDS), intraventricular hemorrhage (IVH), bronchopulmonary dysplasia (BPD), necrotizing enterocolitis (NEC), and periventricular leukomalacia (PVL) were based on the latest updated diagnostic criteria in the standard textbook of neonatology [20]. Shock was defined as a mean blood pressure<lower limit according to gestational age that was unresponsive to fluid treatment or required vasoactive agents [21]. Sepsis attributable mortality was defined as a patient who expired within 7 days after onset of sepsis, those who died of infectious complications or clinically progressive deterioration since BSI.

The adequacy of antimicrobial therapy were determined at three time points: empiric antibiotic therapy at the time of initial culture obtained, when the blood culture preliminary revealed a positive result and only Gram stain result was available, and when the final identification and susceptibility results of all pathogens were available. We also evaluated the effect of available preliminary results on the modification of antimicrobial regimens (eg, broadening or narrowing the spectrum of coverage).

### Statistical methods

The case and control episodes were compared to evaluate the differences regarding to microbiology, clinical characteristics, treatment, and outcomes. Categorical data were compared with χ^2^ test or Fisher's exact teat, and tested for odds ratios (OR). We compared normally distributed continuous variables via Student's *t* test and used the Mann-Whitney *U* test for nonparametric continuous factors. All *P* values were two tailed, and *P* values<0.05 were considered to be statistically significant. Statistical analyses were performed using SPSS version 15.0 (SPSS®, Chicago, IL).

## Results

### The epidemiology and microbiology of neonatal PBSI episodes

During this study period, a total of 1017 episodes of neonatal BSIs in 773 neonates were identified. Forty-five episodes of PBSI (4.4% of all neonatal BSIs) occurred in 43 neonates were defined as case episodes. The majority of PSBIs were caused by two bacterial pathogens (43/45, 95.6%), and only two PSBI episodes had three pathogens identified. The majority of PBSIs had bacteria identified from a single blood culture, and only two PBSIs episodes had bacteria identified from different blood specimens within a 48-hour period. Given the overall cohort of 5010 neonates surviving for more than five full days of life in our NICU during the study period and over a total of 253,655 neonatal-hospital days, the incidence rate of PBSI in our NICU is 1.77 case episode per 10,000 neonate-hospital days.

**Figure 1:**
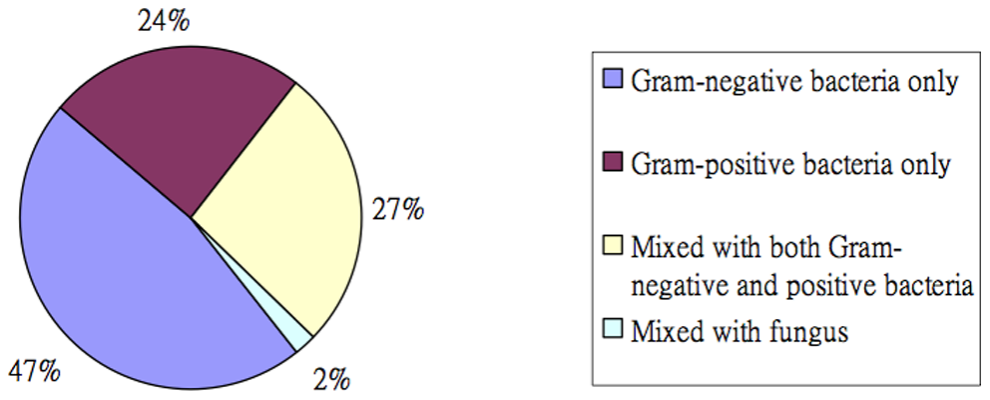
Microbiological categories of organisms identified among patients with polymicrobial bloodstream infections.

The microbiology of PBSI differed from monomicrobial infections (Table 1). Gram-negative organisms constituted nearly three-fifth (59.8%) of the case group organisms, which was a significantly higher percentage than Gram-positive organisms (59.8% vs. 39.1%, *P* = 0.003). Gram-negative organisms were also more common in case episodes than the controls (OR: 2.83, *P*<0.001). In 33 (73.3%) episodes of PBSIs, at least one of the causative organisms was Gram-negative bacilli, and 22 (48.9%) episodes of PBSIs were caused by two or more Gram-negative organisms (figure 1). Only one PBSI episode had *Candida* spp. grown in the blood culture.

**Table 1:** Pathogens isolated in 45 cases of polymicrobial bloodstream infection and 90 controls.

Pathogens	Case (%), total n = 45	Control (%), total n = 90	Odds Ratio	*P* value
	total microorganisms = 92			
**Gram-positive organism**	36/92 (39.1)	54/90 (60)	0.448	0.008
Coagulase-negative *Staphylococcus*	16/45 (35.6)	37/90 (41.1)	0.790	0.534
*Staphylococcus aureus*	8/45 (17.8)	11/90 (12.2)	1.553	0.384
*Enterococcus* species	11/45 (24.4)	4/90 (4.4)	6.956	0.002
*Group-B streptococcus*	0/45 (0)	2/90 (2.2)	NA	-
Others	1/45 (2.2)	0/90 (0)	NA	-
**Gram-negative organism**	55/92 (59.8)	31/90 (34.4)	2.829	<0.001
*Klebsiella* spp.	16/45 (35.6)	11/90 (12.2)	3.962	0.002
*Escherichia coli*	11/45 (24.4)	8/90 (8.8)	3.316	0.018
*Enterobacter* spp.	14/45 (31.1)	4/90 (4.4)	9.710	<0.001
*Pseudomonas aeruginosa*	1/45 (2.2)	2/90 (2.2)	NA	1.000
*Acinetobacter baumannii*	6/45 (13.3)	4/90 (4.4)	3.308	0.076
*Serratia marcescens*	2/45 (4.4)	1/90 (1.1)	4.140	0.251
Others*	5/45 (11.1)	1/90 (1.1)	11.125	0.030
ESBL-producing bacteria	7/45 (15.6)	4/90 (4.4)	3.961	0.036
**Fungus**	1/45 (2.2)	5/90 (5.6)	0.386	0.392

ESBL: extended-spectrum β-lactamase, NA: not assayed.

Including *Citrobacter freundii* (2), *Stenotrophomonas maltophilia* (1), *Morganella morganii* (1), and *Preteus mirabilis* (1) in the case episodes.

Specific bacteria more commonly involved in case episodes included *Enterococcus* spp. (OR: 6.96, *P* = 0.002), *E. coli* (OR: 3.32, *P* = 0.018), *Klebsiella* spp. (OR: 3.96, *P* = 0.002), *Enterobacter* spp. (OR: 9.71, *P*<0.001), and other rare Gram-negative organisms (OR: 11.13, *P* = 0.030). There were also more ESBL-producing bacteria in the case episodes than the controls (OR: 3.96, *P* = 0.036).

### Antimicrobial therapy in PBSI

All case episodes were administrated with combined antibiotics for coverage of both Gram-positive and Gram-negative bacteria initially. Empiric antifungal therapy was instituted for three case episodes, and anti-anaerobes (metronidazole) was given in another three cases. Empiric antimicrobial therapy was considered adequate to treat all pathogens of PBSIs in 29 (64.4%) episodes. The adequacy of empiric antimicrobial therapy was comparable between the PSBI cases and the controls (64.4% vs. 73.3%, *P* = 0.321). In 12 PBSI episodes of mixed Gram-positive and Gram-negative bacteria, preliminary Gram stain results showed Gram-negative (n = 11) or Gram-positive (n = 1) infection only, which misled the clinicians to narrowing the antimicrobial coverage in seven cases. From blood cultures obtained to final identification of all bacteria of PBSIs, 31 (68.9%) cases had modification of antimicrobial regimens, among which 16 (35.6%) required broadening of antimicrobial coverage. All patients were treated with adequate antimicrobial therapy after complete identification and susceptibilities were available. The median time to receive adequate antimicrobial coverage for inadequately treated PBSIs was 59 hours (range: 43–94 hours) from illness onset.

### Clinical comparisons between case and control episodes

The 45 episodes of PBSI were matched with 90 episodes of monomicrobial BSI. Potential risk factors for PBSI are shown in Table 2. Given the matched case-control design, most demographics were comparable between the two groups. PBSIs were more likely to occur as a recurrent episode of BSI in case patients than the control patients were (31.1% vs 14.4%, *P* = 0.038). Infants with PBSIs were more likely to have an endotracheal tube in place (55.6% vs. 31.1%, *P* = 0.009) and underlying GI pathology (15.6% vs. 3.3%, *P* = 0.011).

**Table 2:** Demographics and potential risk factors for polymicrobial bloodstream infection by comparisons with monomicrobial bloodstream infection.

Variables	Cases (%)	Controls (%)	P value
Gestational age (weeks), median (IQR)	30.0 (27.5–36.0)	30.0 (26.0–35.0)	0.581
Birth body weight (g), median (IQR)	1355.0 (807.0–2062.0)	1382.5 (800.0–2020.0)	0.946
Male gender	21 (46.7)	49 (54.4)	0.466
Low apgar score at 5 minutes (≤7)	19 (42.2)	30 (33.3)	0.346
Method of delivery (Cesarean section)	26 (57.8)	42 (46.7)	0.274
Outborn	11 (24.4)	34 (37.8)	0.175
Day of bloodstream infection onset (day), median (IQR)	31.0 (14.0–71.5)	25.5 (17.0–45.3)	0.438
Episode sequence of bloodstream infection			0.038
1^st^ episode	31 (68.9)	77 (85.6)	
Recurrent episode	14 (31.1)	13 (14.4)	
Previous operation (within one month)	12 (26.7)	14 (15.6)	0.164
Use of steroid (within one month)	5 (11.1)	8 (8.9)	0.680
Exposure to broad-spectrum antibiotics¶ (within one month)	12 (26.7)	36 (40.0)	0.181
Any underlying chronic conditions*	28 (62.2)	49 (54.4)	0.462
Congenital anomalies	4 (8.9)	8 (8.9)	1.000
Neurological sequelae or comorbidities	6 (13.3)	8 (6.9)	0.550
Bronchopulmonary dysplasia	19 (42.2)	30 (33.3)	0.346
Chronic GI pathology	7 (15.6)	3 (3.3)	0.011
Others	4 (8.9)	7 (7.8)	0.824
Use of central venous catheter	38 (84.4)	69 (76.7)	0.371
Duration of central venous catheter placement (day), median (IQR)	18.0 (12.5–24.0)	17.0 (11.0–23.5)	0.241
On high frequency oscillatory ventilator	5 (11.1)	3 (3.3)	0.074
Under invasive ventilation (intubation)	25 (55.6)	28 (31.1)	0.009
Use of total parenteral nutrition/intrafat	29 (64.4)	50 (55.5)	0.349

Indicating the presence of chronic conditions at onset of bloodstream infection, some cases may have more than one chronic condition.

Broad-spectrum antibiotics included vancomycin, teicoplanin, 3^rd^ and 4^th^ generation cephalosporin, and carbapenem.

Clinical characteristics of PBSI and control episodes are listed in Table 3. Clinical presentations of infants with PBSIs were similar to those with monomicrobial BSIs in terms of fever and apnea, bradycardia and cyanosis. Case patients with PBSIs were significantly more likely to meet criteria of severe sepsis or septic shock (26.7% vs. 7.8%, *P* = 0.007), and significantly more often to have coagulopathy, DIC and gastrointestinal symptoms (*P* = 0.003, 0.001, and 0.040, respectively). Using NTISS to score the severity of illness, case patients had a significantly more severe degree of illness (mean score 17.8±4.9 vs. 15.3±4.0, *P* = 0.004). Because recurrent bacteremia was not often present, symptoms attributable to infection were prolonged in case episodes when compared with controls, including a significantly prolonged feeding intolerance (>3 days) and hematologic abnormality, although the rates of persistent bacteremia were comparable. There was also a trend in the case patients toward longer hospital stay (84.0 days vs. 72.9 days) and higher sepsis-attributable mortality rate (13.3% vs. 4.4%) although the differences did not meet statistical significance (p = 0.063). However, case patients had a significantly higher in-hospital mortality rate (20% vs. 7.8%, p = 0.049).

**Table 3:** Clinical presentation, clinical course and outcomes of polymicrobial bloodstream infection versus monomicrobial bloodstream infection.

Clinical characteristics	Cases	Controls	Odds ratio (95% CI)	P value
Clinical presentations				
Fever (>38.0°C)	27 (60.0)	40 (44.4)	1.87 (0.91–3.88)	0.102
Apnea, bradycardia and cyanosis	29 (64.4)	55 (61.1)	1.23 (0.57–2.61)	0.704
Abdominal distension and feeding intolerance	31 (68.9)	45 (50.0)	2.33 (1.08–5.03)	0.040
GI bleeding and/or coagulopathy	18 (40.0)	14 (15.6)	3.62 (1.59–8.26)	0.003
Disseminated intravascular coagulopathy	10 (22.2)	4 (4.4)	6.14 (1.81–20.90)	0.001
Severe sepsis or septic shock	12 (26.7)	7 (7.8)	6.18 (2.02–18.91)	0.007
Clinical course				
Persistent bacteremia*	4 (8.8)	5 (5.6)	1.66 (0.42–6.51)	0.464
Persistent ileus and/or feeding intolerance >3 days	22 (48.9)	25 (27.8)	2.56 (1.21–5.42)	0.020
Hematological abnormality#	30 (66.7)	34 (37.8)	3.29 (1.55–6.99)	0.002
NTISS (mean ± SD)	17.8±4.9	15.3±4.0	-	0.004
Outcomes				
Removal of central venous catheter	20/38 (52.6)	31/69 (44.9)	1.44 (0.65–3.20)	0.421
Sepsis attributable mortality	6 (13.3)	4 (4.4)	3.31 (0.88–12.39)	0.063
Overall in-hospital mortality	9 (20.0)	7 (7.8)	2.96 (1.03–8.58)	0.049
Length of hospitalization (median, IQR)	84.0 (52.5–122.75)	72.0 (37.8–112.3)	-	0.275
Recurrent bloodstream infection within one month	11 (24.4)	17 (18.9)	1.39 (0.59–3.29)	0.502

All data were expressed as number (percentage %), unless indicated otherwise.

Defined as two or more consecutive positive blood cultures with at least 48 hours apart.

^#^ Indicated requirement of any blood transfusion after bloodstream infection onset.

NTISS: Neonatal Therapeutic Intervention Scoring System, BSI: bloodstream infection, SD: standard deviation, DIC: disseminated intravascular coagulopathy, IQR: interquartile range.

## Discussion

Results from this study showed that, polymicrobial BSIs accounted for 4.4% of all neonatal BSIs. We found a significantly higher percentage of Gram-negative bacilli as the causative pathogens of PBSIs than monomicrobial BSIs and overall neonatal BSIs. PBSIs in neonates were significantly associated with higher severity of sepsis and a higher rate of persistent clinical symptoms, but not associated with a significantly higher rate of sepsis-attributable mortality rate (13.3% vs. 4.4%, p = 0.063). Inconsistent with previous studies which concluded underlying chronic conditions or GI pathology as the predisposing factors of PBSIs [1], [2], we found more than one-third of PBSIs can occur in neonates without any underlying conditions, while the presence of mechanical ventilator was significantly associated with PBSI. Owning to a high variety of pathogens involving in PBSIs, broadening of antimicrobial therapy was often required in neonates with PBSIs.

Bacterial pathogens identified for PBSIs in the present study were different from those in previous reports [1], [2], [12], and Gram-negative bacilli were more common identified. These may be due to the different criteria of enrolling case episodes of PBSIs. For example, due to the strict inclusion criteria of CoNS in PBSIs in our study, the prevalence rate of PBSIs among all BSIs in our NICUs was relatively lower than that in previous studies [1]–[7], [12]. In contrast, *Enterococcus* spp. were among common pathogens involved in PBSIs in this study and was associated with GI pathology, which were consistent with previous reports [22]–[24].

An important strength in this report is the selection of controls, which were based on the pathogen distributions of overall neonates with BSIs. Therefore our controls were more able to represent the overall BSIs cohort. There may be selection bias in Bizzarro et al's match of only birth date, gender and weight to investigate the impact of PBSIs on outcome and mortality, since the bacterial pathogens are well known to be the independent factors of outcomes [14]–[16].

Our study did not show statistically significant differences with regard to the presence of underlying chronic conditions and duration of indwelling CVC between PBSIs and controls, which were the findings of Bizzarro *et al* [2]. However, the median duration that the CVC had been in place in our case and control groups were both comparable with our previous findings that catheter-related BSIs often occurred after longer indwelling time of CVC (a median of 18 days after CVC insertion) [25], [26]. The significantly shorter duration of CVC in monomicrobial BSIs in Bizzarro's study (median 9.8 days) is unusual and may be due to intercenter variation. There have been inconsistent conclusions with regarding to which neonates or children are at risk of PBSI [1]–[3], [12], [13]. As a matter of fact, the definition of chronic conditions in the NICU is debatable and there is no universal agreement in scoring the severity. Besides, the status of neonatal chronic conditions, such as BPD or secondary pulmonary hypertension, is changeable and maybe improved over time, which potentially leads to difficulties in qualification.

Our data were consistent with previous studies that PBSIs were associated with higher severity of illness [1], [3], [12], although the final outcomes or mortality rates were proven insignificantly different. The initial adequacy of empiric antibiotics was comparable between PBSIs and controls, but significantly more modification or expansion of antimicrobial regimens were encountered in the PBSIs. A large proportion of our PBSIs were informed as monomicrobial BSI initially, which misled the clinicians in the decisions of antimicrobial regimens. Besides, more than half inadequacy of empiric antibiotics in the control group was due to delay administration of vancomycin or teicoplanin for CoNS or *S. aureus* BSIs (13/24, 54.2%), which were less likely to cause serious consequences in our cohort. The delay of adequate antimicrobial therapy to cover all the pathogens of PBSIs undoubtedly accounted for the more severe illness and prolonged septic symptoms than the controls. A possible explanation we failed to document the higher rate of persistent bacteremia in our PBSI group was due to repeat blood culture drawing was not often done in our NICU.

There are some limitations of our study. Although our data came from the largest NICU center in Taiwan, the small study size of PBSIs were unable to detect a difference in outcomes and potential risk factors. Therefore we did not solve the problem which patients are at risk of PBSIs. Since some infants enrolled had multiple episodes of PBSI and were calculated accordingly without adjustment, the analysis and interpretation of risk factors for PBSI should be meticulous. The character of data from single center also limits the generalizability to other geographical areas or institutions. Finally, given the retrospective nature of this study, some unmeasured confounding factors may exist, including the control cases matching, the recognition of multiple-organism BSI by attending physicians and the factors that influenced their decisions in modifying antibiotics.

In conclusion, in view of a neonate with PBSI is possibly to have more severe clinical illness, it is mandatory to direct multiple broad-spectrum antibiotics to cover both Gram-positive and Gram-negative bacteria before final identification of all organisms and susceptibilities are informed. Neonates with PBSI also had a longer hospital stay, a higher sepsis-attributable mortality rate, although not statistically significant, and had a significantly higher in-hospital mortality rate.
